# Renoprotective and Oxidative Stress-Modulating Effects of Taxifolin against Cadmium-Induced Nephrotoxicity in Mice

**DOI:** 10.3390/life12081150

**Published:** 2022-07-29

**Authors:** Abdulmohsen I. Algefare

**Affiliations:** Department of Biological Sciences, Faculty of Science, King Faisal University, Al-Ahsa 31982, Saudi Arabia; aalgefare@kfu.edu.sa

**Keywords:** TA, cadmium, nephrotoxicity, Nrf2, apoptosis, antioxidants

## Abstract

Cadmium (Cd) is an inessential trace metal that accumulates in the kidney and may lead to renal toxicity by mediating oxidative stress (OS), inflammatory reactions, and apoptosis. The main objective of this experiment was to inspect the protecting potential of taxifolin (TA) on Cd-induced renal toxicity. Adult male mice were allocated into equal five groups as follows: control, TA-treated (50 mg/kg, oral), CdCl_2_-treated (4 mg/kg body weight (BW), p.o.), pretreated with TA (25 mg/kg) 1 h before CdCl_2_ injection (4 mg/kg BW, p.o.), and pretreated with TA (50 mg/kg) 1 h before CdCl_2_ injection (4 mg/kg BW, p.o.) for 14 days. Cd-intoxicated mice revealed higher serum urea and creatinine levels and notable histopathological alterations in the renal tissues. Malondialdehyde (MDA), nitric oxide (NO), nuclear factor-kappa B (NF-κB) p65, tumor necrosis factor-α (TNF-α), and IL-1β were increased. In contrast, glutathione levels, catalase and superoxide dismutase activities, and IL-10 levels were decreased under Cd-administered effects. Conversely, the TA pre-treatment highly protected tissues from Cd-toxicity, improved renal function, decreased MDA and NO levels, attenuated inflammation, and improved redox status in the renal tissues of Cd-intoxicated mice. The TA pre-treatment of Cd-intoxicated mice showed down-regulation of both Bax and caspase-3 protein and up-regulation of Bcl-2 protein expression in the kidney. Furthermore, TA pre-treatment induced higher upregulation of nuclear factor erythroid 2-related factor 2 (Nrf2) and heme oxygenase 1 (HO-1) expression in kidney cells of Cd-intoxicated mice. Therefore, TA can protect renal tissues against Cd-induced nephrotoxicity via improving redox status, modulating inflammation, diminishing cell apoptosis, and activating the Nrf2/HO-1 signaling pathway.

## 1. Introduction

Cadmium (Cd) is a common ecological contaminant poisonous to several human organs, including the kidney, heart, testis, liver, and lung [1,2]. Several studies have shown that prolonged Cd exposure causes renal toxicity [3,4,5]. Metal mining, refining, smelting, fossil fuel burning, and rubbish burning all contribute to prevalent Cd contamination [6]. Furthermore, feedstuffs, drinking water, inspired cigarette smoke or polluted air, and absorption of polluted soil and dust created by people and animals that have earlier collected Cd in their biological cells are also causes of Cd exposure [7]. Indeed, Cd is a non-biodegradable, poisonous element with a prolonged biological half-life and is harmful to plants, humans, and animals [8]. Several attempts by national organizations and government agencies to limit Cd usage have been applied. However, a common human health hazard persists, mainly in third-world countries where ecological rules are either insufficient or disregarded [9].

Despite low concentrations of Cd subjection in the renal tissue, it accumulates in the tubular epithelium in the form of a Cd-metallothionein complex, leading to proximal tubular dysfunction [8]. Several experiments have shown that Cd-stimulated renal toxicity is mediated by the liberation of Cd-metallothionein compounds from impaired Kupffer cells, which are refined by the glomerulus, endocytosed through the proximal tubular cells, and destroyed by lysosomes after Cd accumulation [10]. Cd exposure or accumulation is well documented to overproduce reactive oxygen species (ROS) and reveal inflammation, eventually culminating in kidney cell apoptosis [3]. Therefore, activation of cytoprotective pathways, e.g., nuclear factor erythroid 2-related factor 2 (Nrf2) and heme oxygenase 1 (HO-1) and attenuation of oxidative stress (OS), consequent inflammation, and apoptosis can represent an important strategy to prevent Cd-induced nephrotoxicity.

Indeed, several natural products are beneficial in eliminating Cd-induced nephrotoxicity [11,12,13,14]. Taxifolin (TA) is a flavonoid substance present in Siberian larch (*Larix sibirica*) as well as being categorized as a metal-chelating agent [15]. In addition, it is a non-mutagenic agent and has reduced toxicity compared to other flavonoids [16]. Moreover, it has a range of biological actions involving antioxidant, anti-inflammation, and antiapoptotic features [17,18]. TA attenuated cisplatin-induced pulmonary destruction in rats through modulation of OS and restoration of antioxidants [19]. In addition, TA attenuated renal injury and fibrosis in unilateral ureteral obstruction (UUO) animal models by alleviating OS, inflammation, TGF-β1, Smad-2, α-SMA, CTGF, and collagen type I in rats [20]. TA has also been shown to prevent OS-stimulated retinal pigment epithelium (RPE) cell destruction and apoptosis via activation of Nrf2 and the phase II antioxidant enzyme system. Furthermore, TA ameliorated acute alcohol-stimulated hepatic damage via modulating the NF-κB-mediated inflammation in mice [21].

To our knowledge, no studies have been published on TA’s preventive impact on Cd-induced renal toxicity in mice. Thus, this research was adopted to explore the possible protecting impact of TA versus Cd-induced kidney damage, mentioning its power to attenuate OS, inflammation, and apoptosis, and augment Nrf2/HO-1 signaling.

## 2. Materials and Methods

### 2.1. Animals and Treatment

Procedures, including the usage of mice, were conducted in line with the rules of the National Institutes of Health (NIH publication No. 85–23, revised 2011) and were accepted by the research ethics Committee at King Faisal University (KFU-REC-2022-MAY-EA00637, Al Hofuf, Saudi Arabia). Adult male mice were employed in this experiment. Mice were reared in typical cages at normal atmospheric conditions (25 ± 2 °C) with a 12 h light/dark cycle and were allowed feed and water ad libitum. Post acclimatization for 7-days, the mice were allocated into five groups (N = 6) as follows: Group I: untreated control group, Group II: TA-treated (50 mg/kg, p.o.) group [19,22], Group III: CdCl_2_-treated (4 mg/kg BW, p.o.) group [14], Group IV: pretreated with TA (25 mg/kg) 1 h before CdCl_2_ (4 mg/kg BW, p.o.) group, and Group V: pretreated with TA (50 mg/kg, p.o.) 1 h before CdCl_2_ group (4 mg/kg BW, p.o.) for two weeks [14].

TA (purity > 95%) was obtained from Biosynth Carbosynth (Newbury, UK). TA was dissolved in 0.5% DMSO and given orally via oral gavage [23]. CdCl_2_ (4 mg/kg body weight, p.o. via oral gavage) was dissolved in distilled water containing 1% tween 80 [14]. The TA dose was determined based on previous reports showing TA’s antioxidant and anti-inflammatory effects in vivo [19,24,25].

### 2.2. Estimation of Serum Renal Function Markers

The levels of serum urea and creatinine were determined by employing kits supplied by Spinreact (Girona, Spain), applying the manufacturer’s guidelines.

### 2.3. Estimation of Malondialdehyde (MDA), Nitric Oxide (NO), and Antioxidants

Renal MDA and NO levels were measured in renal tissues following the procedures defined by Ohkawa et al. [26] and Green et al. [27], respectively. The superoxide dismutase (SOD) [28] and catalase (CAT) [29] activities and reduced glutathione (GSH) [30] contents were determined in the renal tissues. The HO-1 content of renal tissue was measured by applying a special ELISA kit (MyBioSource, San Diego, CA, USA) after the manufacturer’s instructions [31].

### 2.4. Assessment of Inflammatory Cytokines in the Renal Tissue

Renal levels of tumor necrosis factor-alpha (TNF-α) and interleukin-1beta (IL-1β) were estimated by ELISA kits provided by R&D Systems (Minneapolis, MN, USA). IL-10 level was determined using an ELISA kit procured from MyBioSource (San Diego, CA, USA). All assays were performed according to the manufacturer’s instructions.

### 2.5. Histological Examination of Kidney Sections

Kidney samples were kept in 10% buffered formalin, inserted in paraffin, and sliced into 5 μm sections. Paraffin-embedded tissues were then deparaffinized by variations in xylene and rehydrated to reduce ethanol levels. The sections were subjected to hematoxylin and eosin (H&E). Histological evaluation of kidney tissue injury was performed in a blinded manner.

### 2.6. Immunohistochemistry

By immersing in xylene and descending graded concentration of ethanol solutions, paraffin-embedded sections were sliced, deparaffinized, and hydrated before being microwave antigen retrieving treated. After that, a 0.3 % hydrogen peroxide per methanol solution was utilized to stop endogenous peroxidase activity. The prepared slides were cooled at ambient temperature, and nonspecific binding was blocked with normal serum for 20 min at ambient temperature. Then, they were blended with anti-Bax, anti-caspase 3, anti-Nrf2, anti-Bcl-2 (all purchased from Invitrogen, Waltham, MA, USA), and anti-NF-κB p65 (purchased from Santa Cruz Biotechnol., Dallas, TX, USA) and preserved 24 hrs in the refrigerator at 4 °C. The 2^ry^ antibodies were smeared after washing the slides with PBS several times. The color expansion was prompted via incubation with 3,3′-diaminobenzidine-tetrahydrochloride-H_2_O_2_ solution, and then all prepared sections were stained with Mayer’s hematoxylin and examined by light microscopy. Staining intensity was estimated and achieved as a positive expression% in 1000 cells per 8 HPF for NF-ĸB p65, caspase 3, and Bax, while Nrf2 and Bcl-2 immunostaining was verified via the zone of + ve expression by applying ImageJ evaluation software (NIH, Bethesda, MD, USA).

### 2.7. Statistical Evaluation

All calculated data were described as mean ± standard error of the mean (SEM). All statistical comparisons between trial groups were computed using GraphPad Prism 7 software (San Diego, CA, USA) and one-way ANOVA combined with Tukey’s post-hoc test for multiple comparisons. A *p*-value < 0.05 was deemed significant.

## 3. Results

### 3.1. TA Prevents Cd-Induced Kidney Damage

**Figure 1:**
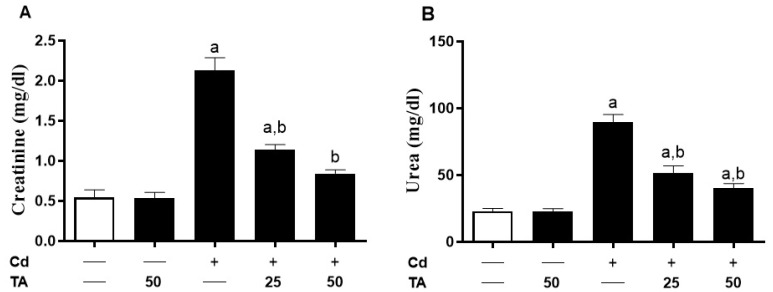
Effects of TA on renal function markers in Cd-intoxicated mice. TA reduced (**A**) creatinine and (**B**) urea concentrations in serum of Cd-intoxicated animals. Data are mean ± SEM, (*n* = 6). a *p* < 0.05 vs. control, while b *p* < 0.05 vs. Cd.

**Figure 2:**
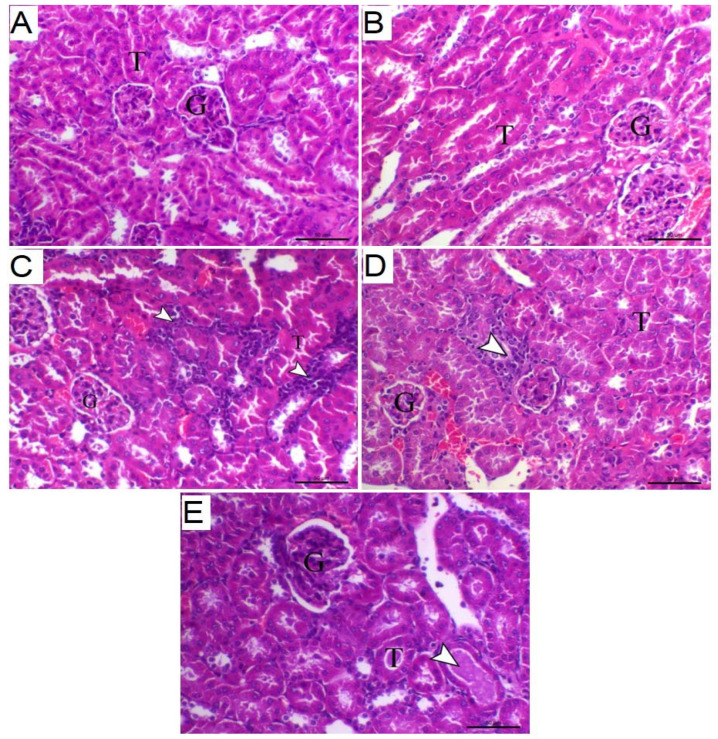
Effect of TA against Cd-induced histological changes in kidney of mice. Photomicrographs of kidney sections from (**A**) control and (**B**) TA-administered mice revealing normal renal glomeruli (G) and tubules (T); (**C**) Cd-intoxicated mice representing revealing features of interstitial nephritis associated with marked mononuclear inflammatory cells infiltration (arrowheads) (G indicates glomeruli and T indicates tubules); (**D**) Cd-intoxicated mice pre-treated with 25 mg TA demonstrated marked decrease in interstitial nephritis with marked decrease mononuclear inflammatory cells infiltration (arrowheads) (G indicates glomeruli and T indicates tubules); and (**E**) Cd-intoxicated mice pre-treated with 50 mg TA showing marked decrease the inflammatory lesions inside the renal parenchyma with mild protein inside the lumen of few renal tubules (arrowhead) (G indicates glomeruli and T indicates tubules) (H&E, X 200, bar = 50 µm).

The impact of TA against Cd nephrotoxicity was assessed by assessing kidney function markers (Figure 1A,B) and histopathological changes (Figure 2). Kidney impairment was shown in Cd-intoxicated mice by a remarkable (*p* < 0.05) increase in creatinine (Figure 1A) and urea (Figure 1B) serum concentrations. The TA pretreatment had no influence on these markers in normal mice. In contrast, pretreatment of Cd-intoxicated mice with TA reduced urea and creatinine levels (*p* < 0.05) compared to normal levels.

The histopathological examination of the renal tissue sections from control and TA-treated mice showed normal tissue features with normal renal tissue structure. The Cd-intoxicated mice demonstrated features of interstitial nephritis associated with marked mononuclear inflammatory cells infiltration. When Cd-intoxicated mice were pre-treated with TA, all observed alterations were significantly reduced (Figure 2).

### 3.2. TA Attenuates Cd-Induced Renal Oxidative Stress

**Figure 3:**
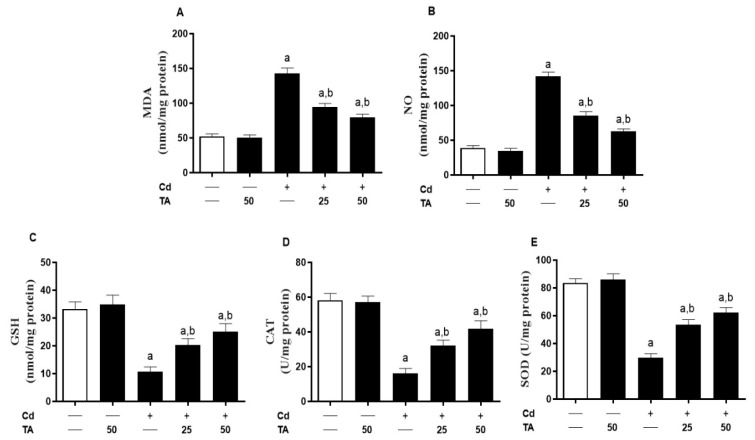
Impacts of TA treatment on the oxidative state of the renal tissue in Cd-intoxicated mice. The TA pre-treatment of Cd-intoxicated mice decreased kidney (**A**) MDA and (**B**) NO contents and stimulated (**C**) GSH, (**D**) CAT, and (**E**) SOD levels. Results are mean ± SEM, (*n* = 6). a *p* < 0.05 vs. control, while b *p* < 0.05 vs. Cd.

Because OS is thought to be a common contributor to Cd-provoked renal damage, we investigated the effect of TA on renal redox status. The MDA (Figure 3A) and NO (Figure 3B) levels in mice subjected to Cd were increased significantly (*p* < 0.05) as contrasted with the control. Moreover, the kidney GSH (Figure 3C) contents and CAT (Figure 3D) and SOD (Figure 3E) activities were significantly (*p* < 0.05) lowered in Cd-intoxicated mice. TA pretreatment prevented all these alterations in Cd-exposed mice. TA did not influence the variables mentioned above in healthy mice.

### 3.3. TA Alleviates Cd-Induced Inflammation in the Kidney

**Figure 4:**
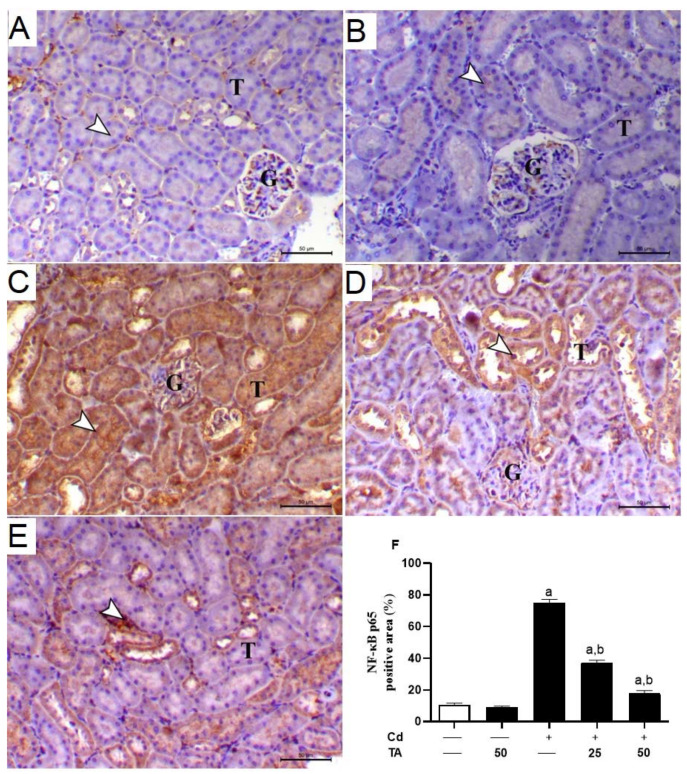
Effect of TA pretreatment on Cd-induced renal inflammation. Photomicrographs of (**A**) control and (**B**) TA-administrated mice revealed slight immunostaining of NF-κB p65 antibody inside the kidney tubular epithelium (arrowhead) (G and T letters indicate renal glomeruli and tubules, respectively); (**C**) Cd-intoxicated mice demonstrated marked immunostaining of NF-κB p65 (cytoplasmic and nuclear) inside the renal tubular epithelium (arrowhead) (G and T letters indicate renal glomeruli and tubules, respectively); (**D**) Cd-intoxicated mice pre-treated with 25 mg TA demonstrated lowering of NF-κB p65 immunostaining inside the renal tubular epithelium (arrowhead) (G and T letters indicate renal glomeruli and tubules, respectively); (**E**) Cd-intoxicated mice pre-treated with 50 mg TA showing the significant lowering of NF-κB p65 immunostaining inside the renal tubular epithelium (arrowhead) (G and T letters indicate renal glomeruli and tubules, respectively) (IHC, X 200, bar = 40 µm); (**F**) Image evaluation of NF-κB p65 immunostaining in renal demonstrated a remarkable elevation in Cd-intoxicated mice and a remarkable reduction in mice pre-treated with TA. Results are mean ± SEM, (*n* = 6). a *p* < 0.05 vs. control, while b *p* < 0.05 vs. Cd.

**Figure 5:**
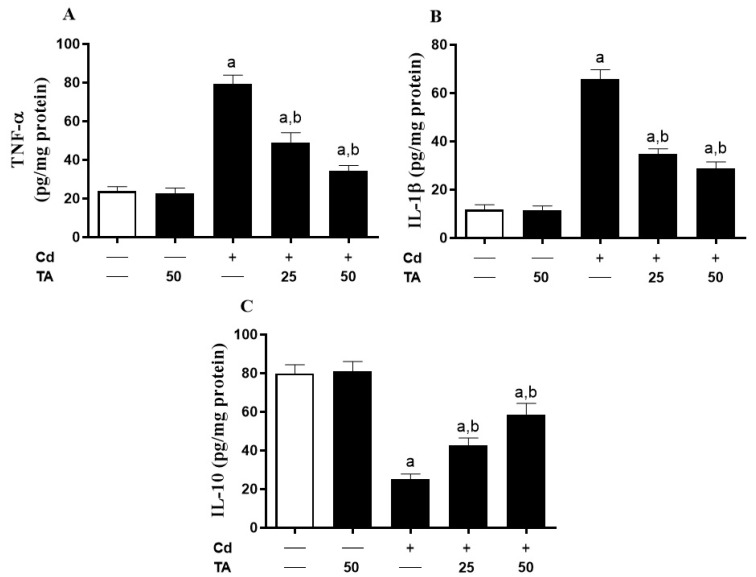
TA reduced (**A**) TNF-α and (**B**) IL-1β and increased (**C**) IL-10 in the kidney of Cd-induced mice. Results are mean ± SEM, (*n* = 6). a *p* < 0.05 vs. control, while b *p* < 0.05 vs. Cd.

TA’s capability to attenuate Cd-induced renal inflammation was assessed by measuring NF-κB p65 expression levels and pro-inflammatory cytokine production in the kidney. When compared with the control group, there was a significant (*p* < 0.05) increase in the renal tissue levels of NF-B p65 (Figure 4A–F) in the Cd-intoxicated mice. Similarly, there was a significant (*p* < 0.05) elevation in TNF-α (Figure 5A) and IL-1β (Figure 5B) levels with a concomitant decline in IL-10 (Figure 5C) levels in the kidney of the Cd-stimulated mice. Conversely, pretreating the Cd-intoxicated mice with TA notably (*p* < 0.05) suppressed the activation of NF-κB p65 expression (Figure 4A–F), likewise concentrations of TNF-α (Figure 5A) and IL-1β (Figure 5B) in the renal tissues. In addition, the TA treatment of Cd-intoxicated mice significantly increased the anti-inflammatory cytokine IL-10 in the kidney (Figure 5C). TA administration had no impact on these pro-inflammatory cytokines in healthy mice.

### 3.4. TA Ameliorates Cd-Induced Renal Apoptosis

**Figure 6:**
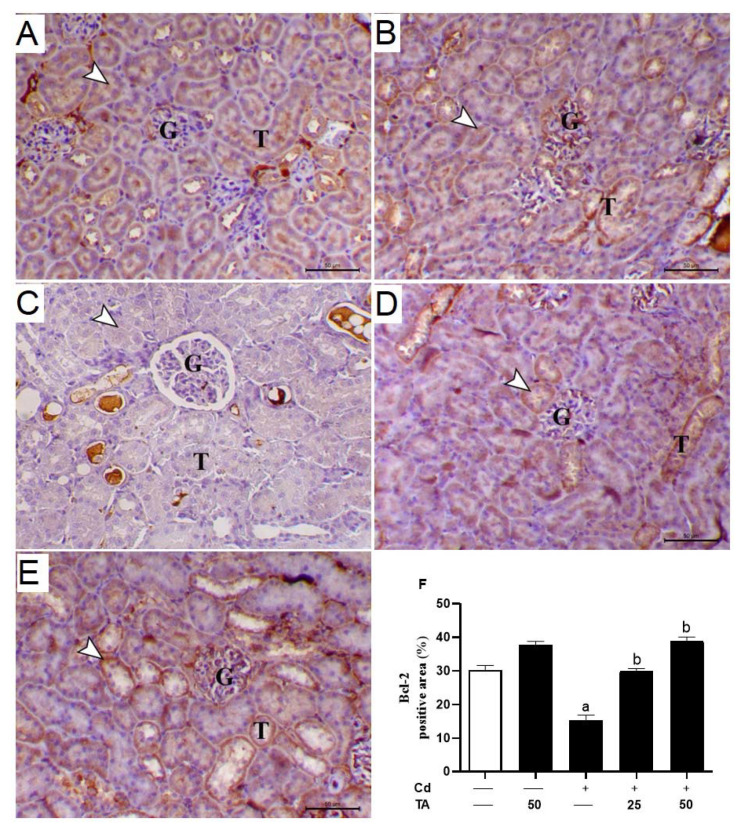
Effect of TA pretreatment on Cd-stimulated decreased renal Bcl-2 expression. Photomicrographs of (**A**) control and (**B**) TA-administrated mice showing marked immunostaining of Bcl-2 antibody inside the renal tubular epithelium (arrowhead) (G and T letters indicate renal glomeruli and tubules, respectively); (**C**) Cd-intoxicated mice demonstrated marked decrease the immunostaining of Bcl-2 antibody inside the renal tubular epithelium (arrowhead) (G and T letters indicate renal glomeruli and tubules, respectively); (**D**) Cd-intoxicated mice pre-treated with 25 mg TA demonstrated a marked increase in immunostaining of Bcl-2 inside the renal tissues (arrowhead) (G and T letters indicate renal glomeruli and tubules, respectively); (**E**) Cd-intoxicated mice pre-treated with 50 mg TA demonstrated a marked increase in Bcl-2 immunostaining inside the renal tissues (arrowhead) (G and T letters indicate renal glomeruli and tubules, respectively) (IHC, X 400, bar = 20 µm); (**F**) Image assessment of Bcl-2 immunostaining in the renal tissue demonstrated a remarkable decrease in Cd-intoxicated mice and a remarkable increase in mice pre-treated with TA. Results are mean ± SEM, (*n* = 6). a *p* < 0.05 vs. control, while b *p* < 0.05 vs. Cd.

**Figure 7:**
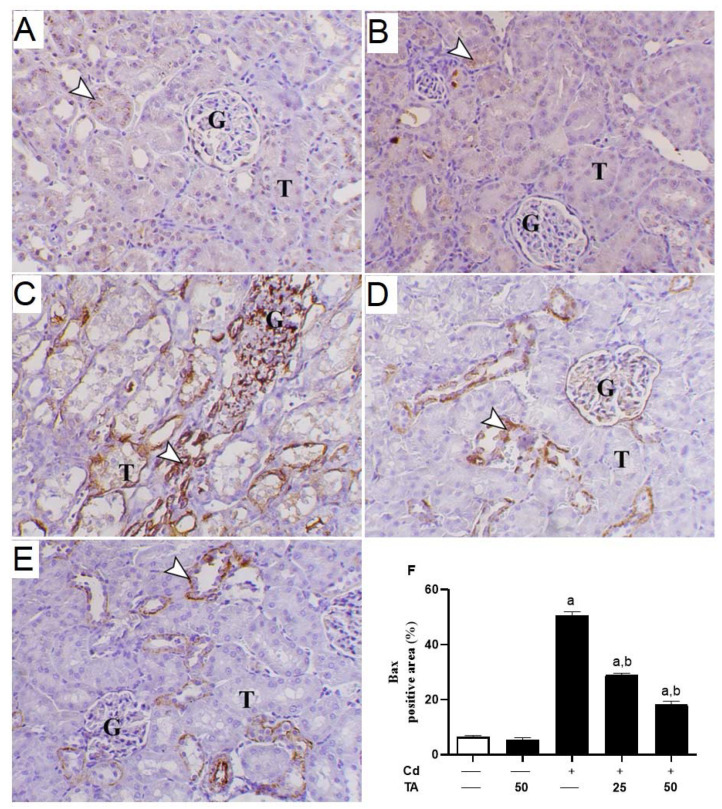
Effect of TA pretreatment on Cd-induced increased renal Bax. Photomicrographs of (**A**) control and (**B**) TA-administrated mice showing mild immunostaining of Bax antibody inside the kidney tubular epithelium (arrowhead) (G and T letters indicate renal glomeruli and tubules, respectively); (**C**) Cd-intoxicated mice demonstrated marked cytoplasmic and nuclear immunostaining of Bax antibody inside the kidney tubular epithelium (arrowhead) (G and T letters indicate renal glomeruli and tubules, respectively); (**D**) Cd-intoxicated mice pre-treated with 25 mg TA demonstrated marked decrease Bax antibody immunostaining inside the renal tubular epithelium (arrowhead) (G and T letters indicate renal glomeruli and tubules, respectively); (**E**) Cd-intoxicated mice pre-treated with 50 mg TA demonstrated marked decrease Bax antibody immunostaining inside the renal tubular epithelium (arrowhead) (G and T letters indicate renal glomeruli and tubules, respectively) (IHC, X 200, bar = 20 µm); (**F**) Image evaluation of Bax immunostaining in the kidney demonstrated a remarkable increase in Cd-intoxicated mice and a remarkable reduction in mice pre-treated with TA. Results are mean ± SEM, (*n* = 6). A *p* < 0.05 vs. control, while b *p* < 0.05 vs. Cd.

**Figure 8:**
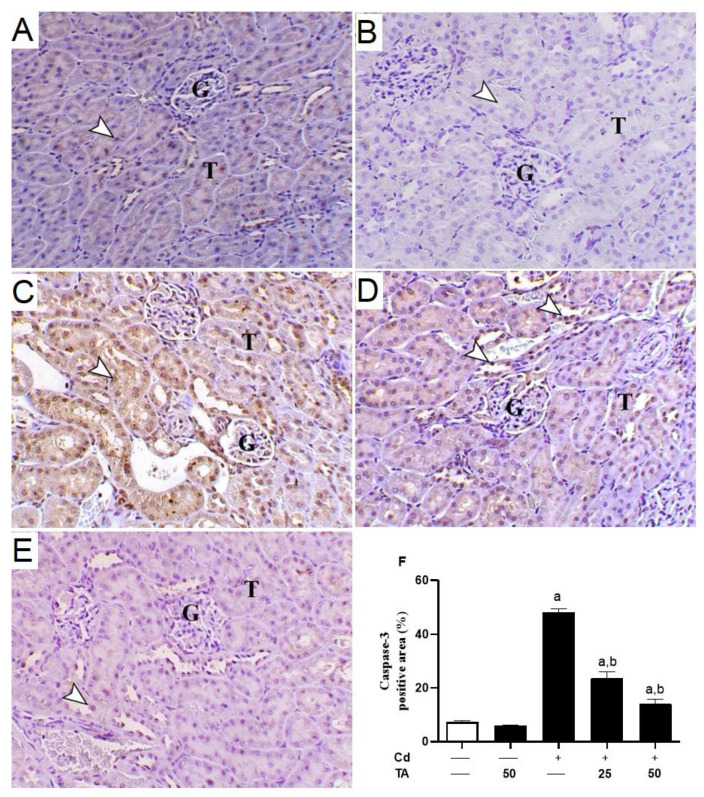
Impact of TA pretreatment on Cd-induced increased renal caspase-3. Photomicrographs of (**A**) control and (**B**) TA-administrated mice revealed mild immunostaining of caspase-3 antibody inside the renal tubular epithelium (arrowhead) (G and T letters indicate renal glomeruli and tubules, respectively); (**C**) Cd-intoxicated mice demonstrated marked immunostaining of caspase-3 (cytoplasmic and nuclear) inside the renal tubular epithelium (arrowhead) (G and T letters indicate renal glomeruli and tubules, respectively); (**D**) Cd-intoxicated mice pre-treated with 25 mg TA demonstrated marked decrease in caspase-3 antibody immunostaining inside the renal tubular epithelium (arrowhead) (G and T letters indicate renal glomeruli and tubules, respectively); (**E**) Cd-intoxicated mice pre-treated with 50 mg TA demonstrated marked decrease in caspase-3 antibody immunostaining inside the renal tubular epithelium (arrowhead) (G and T letters indicate renal glomeruli and tubules, respectively) (IHC, X 200, bar = 40 µm); (**F**) Image evaluation of caspase-3 immunostaining in the renal tissue demonstrated a remarkable elevation in Cd-intoxicated mice and a remarkable reduction in mice pre-treated with TA. Results are mean ± SEM, (*n* = 6). a *p* < 0.05 vs. control, while b *p* < 0.05 vs. Cd.

Increased ROS generation and inflammatory responses significantly contribute to Cd-induced kidney apoptosis. To estimate the impact of TA on Cd-stimulated kidney apoptosis, we assessed the concentrations of kidney Bcl-2, Bax, and caspase-3 expression in mice. In comparison to control mice, the kidney tissue of Cd-intoxicated mice revealed a significant (*p* < 0.05) decrease in Bcl-2 (Figure 6A–F) expression with a concurrent rise in Bax (Figure 7A–F) and caspase-3 (Figure 8A–F). These changes were reduced when Cd-intoxicated mice were pre-treated with TA (Figure 6, Figure 7 and Figure 8). Furthermore, TA alone did not change the above-mentioned variables in healthy mice.

### 3.5. TA Improves Nrf2/HO-1 Signaling Pathway in Renal Tissue

**Figure 9:**
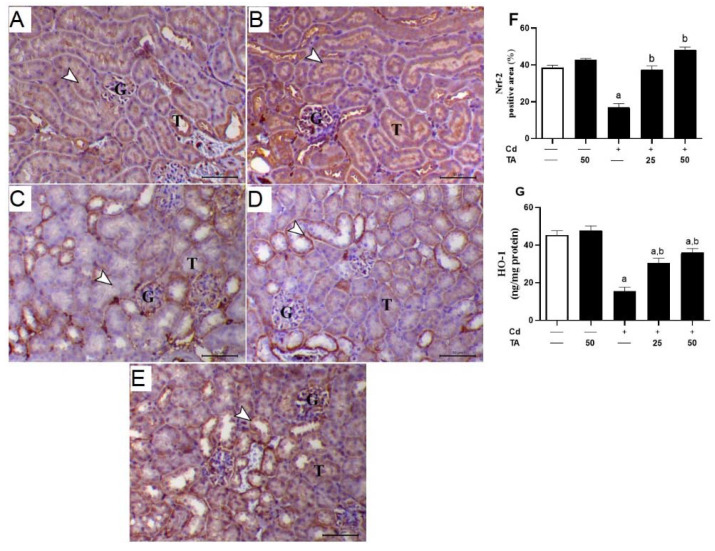
Impact of TA pretreatment on Cd-induced decreased renal Nrf2/HO-1 signaling pathway. Photomicrographs of (**A**) control and (**B**) TA-administrated mice showing marked immunostaining of Nrf2 antibody inside the kidney tubular epithelium (arrowhead) (G and T letters indicate renal glomeruli and tubules, respectively); (**C**) Cd-intoxicated mice demonstrated marked decrease of Nrf2 immunostaining antibody inside the renal tubular epithelium (arrowhead) (G and T letters indicate kidney glomeruli and tubules, respectively); (**D**) Cd-intoxicated mice pre-treated with 25 mg TA demonstrated an increase in Nrf2 immunostaining inside the renal tissues (arrowhead) (G and T letters indicate renal glomeruli and tubules, respectively); (**E**) Cd-intoxicated mice pre-treated with 50 mg TA demonstrated a marked increase in Nrf2 immunostaining inside the renal tissues (arrowhead) (G and T letters indicate renal glomeruli and tubules, respectively) (IHC, X 400, bar = 20 µm); (**F**) Image evaluation of Nrf2 immunostaining in the renal tissue demonstrated a remarkable decrease in Cd-intoxicated mice and a remarkable increase in mice pre-treated with TA; (**G**) TA enhanced renal HO-1 expression in Cd-intoxicated mice. Results are mean ± SEM, (*n* = 6). a *p* < 0.05 vs. control, while b *p* < 0.05 vs. Cd.

Further, to explore TA’s preventive role against Cd nephrotoxicity, the expression degrees of both Nrf2 and HO-1 in the kidney were measured in all experimental groups. As indicated in Figure 9, a significant (*p* < 0.05) decrease in Nrf2 and HO-1 expression levels in the kidneys of Cd-intoxicated mice contrasted with the healthy group. Remarkably, pretreatment of Cd-intoxicated mice with TA markedly up-regulated the renal tissue Nrf2 and HO-1 expression levels. Healthy mice that received TA alone exhibited no changes in the renal Nrf2 and HO-1 expression degrees.

## 4. Discussion

Several recent studies have shown that Cd has carcinogenic and harmful influences on human health because of its abundant contamination of the air, water, and plants [32]. Furthermore, Cd is a well-known harmful industrial contaminant widely discharged and accumulates in many tissues such as the lungs, renal tissue, testis, hepatic tissue, and bones, causing acute organ injury [33]. ROS formation is the important primary mechanism of Cd-induced renal injury because it can alter cell redox balance [34].

Chronic Cd ingestion causes apoptosis and necrosis in the proximal renal tubules, resulting in distinct nephropathy [35]. These data were consistent with our results: Cd administration caused degenerative vacuolar alterations inside the renal tubules and glomerular tuft congestion. Moreover, Aktoz et al. [36] and Fouad and Jresat [37] indicated that administration of 1.2 mg/kg Cd for 30 days might cause proximal tubules and glomeruli dysfunction in rat renal tissue. The renal impairment caused by Cd treatment in mice was shown to be associated with large increases in blood creatinine and urea concentrations. TA treatment of Cd-intoxicated mice restored normal tissue architecture, renal glomeruli, and tubule structure.

Furthermore, pretreatment with TA reduced creatinine and urea serum levels in Cd-intoxicated mice. The improvement in renal structure and kidney function markers shown in our research might be ascribed to TA chelating capabilities and the ability to lower Cd accumulation levels in renal tissue by activating urine clearance [38]. Moreover, TA antioxidant characteristics may be responsible for this improvement by lowering the amounts of ROS in Cd/TA-treated cells compared to Cd-treated cells [14].

Kidney enzymatic and non-enzymatic antioxidant molecules may be the primary endogenous redox defensive system against OS [39]. ROS can damage membrane lipids and may result in increased MDA levels. In addition, NO levels might significantly increase due to oxidative damage to proteins caused by increasing oxygen free radicals in the cell [40]. Moreover, CAT can break down free radical-generated H_2_O_2_ into water and oxygen [41]. In addition, GSH works with glutathione peroxidase (GPx) to decrease peroxides as a co-substitute [42]. Our results indicated that GSH contents and SOD and CAT activities in the renal tissue were significantly reduced in Cd-intoxicated mice. These results were in line with a previous study showing that endogenous antioxidant molecules were reduced significantly after treatment with CdCl_2_ due to its attraction ability for protein-bound thiol groups and Cd ability to increase NADPH oxidase activity [14].

In contrast, treatment of Cd-intoxicated mice with TA restored endogenous antioxidants. Though, experiments exhibited that TA can activate endogenous antioxidant molecules by boosting Cd removal, inhibiting NADPH oxidase, and modulating their upstream signaling pathways [14,38]. Accordingly, recent investigations found that TA improved antioxidant activity and scavenged free radicals [43,44,45]. It is well known that TA has two stabilized aromatic rings, with two hydroxyl groups (–OH) at the meta- and para- positions concerning one other [46]. Moreover, the higher antioxidant properties of TA increased with the presence of -OH groups connected to the aromatic ring [47]. Thus, TA’s potent radical-scavenging feature might depend on its chemical structures and resonance stability of both phenolic rings [48]. Accordingly, TA prevented cisplatin-induced oxidative damage in the lung through modulation of lipid peroxidation and DNA damage and restoration of antioxidants [19].

Increased ROS generation may lead to NF-κB activation and upregulation of its related pro-inflammatory cytokines, aggravating tissue damage [49]. Consistent with several previous studies [13,14,36], Cd-induced NF-κB activation, increased IL-1β, IL-6, and TNF-α, and decreased IL-10 in kidney tissues. The TA pretreatment of Cd-intoxicated mice reduced renal inflammation as evidenced by decreased levels of NF-B p65 and pro-inflammatory cytokines and increased the anti-inflammatory cytokine IL-10 in the renal tissue. IL-10 is an anti-inflammatory cytokine that can inhibit the production of pro-inflammatory cytokines and chemokines and the activation of immune cells. IL-10 attenuates kidney injury in several models of kidney diseases, including lupus nephritis, cisplatin nephrotoxicity, complex immune nephritis, ischemia-reperfusion injury, and transplantation [50]. Salama and Kabel [51] stated that TA lowered iron concentrations and down-regulated the expression of the pro-inflammatory cytokines TNF-α, IL-1β, and IL-6.

Additionally, TA demonstrated a kidney protective effect by inhibiting the binding of nucleotides and the inflammasome’s oligomerization domain-like receptor family pyrin domain-containing 3 (NLRP3) [52]. An additional advantage of TA therapy for Cd-intoxicated patients is supported by the research that shows it might diminish inflammation and/or suppress cytokine storms in those individuals exposed to Cd. Furthermore, a recent study demonstrated that TA showed hepatoprotective effects against alcohol-induced hepatic damage via modulating the NF-κB-mediated pro-inflammatory cytokines production and hepatic inflammation [21].

Indeed, long-term exposure of cells in the renal tubules to ROS overproduction and inflammation may lead to cell death via apoptosis by activating Bax, which promotes the loss of mitochondrial membrane potential [53]. Subsequently, cytochrome c is liberated to the cytoplasm and activates the effector caspase-3 [53], provoking DNA fragmentation, degradation of cytoskeletal proteins, and further release of cytochrome c, leading to cell death [54]. In our study results, Cd-treated animals exhibited a significant down-regulation in Bcl-2. They elevated the expression level of both Bax and caspase-3 genes in renal tissues compared to the untreated group. Consistently, Yuan, et al. [55] found that low-dose Cd exposure may cause kidney apoptosis and eventually impair renal function, which is connected with mitochondrial damage and alterations in degrees of apoptogenic proteins such as Bcl-2, Bax, and caspase-3.

On the other hand, TA pre-treatment prevented Cd-mediated apoptosis in renal cells by down-regulating Bax, up-regulating Bcl-2, and consequently suppressing caspase 3 activations. TA reduced Cd-induced kidney apoptosis by reducing ROS generation, scavenging free radicals, and restoring cellular GPx activity [38]. Previous research has shown that TA may reduce inflammation by inhibiting the NF-κB signaling pathway through ROS suppression [25,56]. Consistently, TA prevents gentamicin-induced apoptotic cell death mediated by the mitochondrial pathway in the kidney [57]. Thus, TA’s anti-inflammatory and anti-apoptotic properties could be related to its ROS-suppressing properties.

To further elucidate the reno-protective effect of TA against Cd-induced nephrotoxicity, we studied the impact of TA pretreatment on the Nrf2/HO-1 signaling pathway in kidney cells. In this study, Cd suppressed renal Nrf2/HO-1 signaling as evidenced by the lowered Nrf2 and HO-1 levels in the kidney, demonstrating the surplus production of ROS. Although activated by ROS under physiological conditions, Nrf2 downregulation has been reported under conditions of excessive and prolonged ROS [31,58,59,60]. Extensive evidence indicates that various naturally occurring compound-mediated Nrf2 activation can protect against drugs/chemicals-induced renal injury [58,59,61]. Several investigations have reported the role of Nrf2 in mediating the protective role of TA [20,62]. Herein, TA upregulated renal Nrf2/HO-1 signaling in Cd-intoxicated mice, as shown by the upregulated Nrf2 and HO-1, which explained the enhanced antioxidant enzymes. Indeed, Nrf2 modulates the response to OS by regulating the expression of numerous genes, including ferritin and HO-1, supported by the antioxidant responses [63]. Accordingly, a previous study showed that TA prevented skin cancer by inducing Nrf2 expression and downstream target genes in JB6 P+ cells via the CpG demethylation process [61]. Furthermore, Xie et al. [62] reported that TA could protect human RPE cells against OS-induced apoptosis via activating the expression of Nrf2 by facilitating its translocation to the nucleus. Collectively, TA effectively protects against Cd-induced kidney injury, perhaps via upregulation of Nrf2/HO-1 signaling.

## 5. Conclusions

The current investigation found that Cd may cause nephrotoxicity by increasing oxidative tissue injury, inflammation, and cell death. Cd-treated mice showed kidney dysfunction associated with noticeable histopathological alterations in the renal tissues. Furthermore, Cd-administered mice had higher MDA, NO, NF-κB p65, TNF-α, and IL-1β concentrations, while GSH and IL-10 levels and CAT and SOD activities were reduced. The TA pre-treatment significantly diminished Cd-mediated nephrotoxicity by suppressing renal OS, inflammatory reaction, and apoptosis and augmenting Nrf2/HO-1 signaling. Thus, TA might be used as an adjuvant against Cd-mediated nephrotoxicity.

## Data Availability

Data analyzed or generated during this study are included in this manuscript.
